# Bio-CM²: Distributed computational optics for cortex-wide cellular imaging

**DOI:** 10.64898/2026.07.27.740823

**Published:** 2026-07-28

**Authors:** Guorong Hu, Qilin Deng, Tianrui Qi, Zhixiong Chen, Bradley C Rauscher, Nathan Chai, Daria Bogatova, Bethany Weinberg, Jesse Smith, Ian G Davison, Martin Thunemann, Anna Devor, Lei Tian

## Abstract

Understanding distributed biological systems, particularly neural circuits, requires simultaneous cellular-resolution imaging across millimeter-scale fields of view (FOV). Existing miniature microscopes remain fundamentally constrained by trade-offs among FOV, spatial resolution, and optical complexity, limiting their ability to bridge cellular microscopy with cortex-scale imaging. Here we introduce distributed computational optics, a framework that distributes image formation across coordinated optical modules and computationally integrates their measurements into a unified image. We realize this framework in Bio-CM², a computational miniature mesoscope that partitions the imaging field across four optical modules while converging their measurements onto a common image sensor. This architecture overcomes the aberration-scaling limitations of conventional miniature optics while avoiding the hardware complexity of multi-camera systems and the contrast degradation associated with optical multiplexing. Bio-CM² achieves a 7.5 × 10 mm² FOV while enabling cellular-resolution *in vivo* imaging at video rates. We demonstrate its utility through two complementary imaging modalities in head-fixed mice: cortex-wide functional vascular imaging, enabling simultaneous quantification of pial arteriole vasomotion and mesoscale hemodynamic functional connectivity, and cellular-resolution calcium imaging, resolving the activity of over 3,000 neurons together with mesoscale neuronal functional connectivity. We further demonstrate the versatility of the platform through cellular-resolution imaging of entire coronal mouse brain sections, population-scale imaging of freely behaving *Caenorhabditis elegans* , and odor-evoked calcium imaging of the main olfactory bulb in head-fixed mice, highlighting its broad applicability across diverse biological systems and imaging modalities. By overcoming the conventional trade-off between FOV and spatial resolution in a compact miniature platform, Bio-CM² establishes distributed computational optics as a scalable framework for multiscale biological imaging.

## Full Text Availability

The license terms selected by the author(s) for this preprint version do not permit archiving in PMC. The full text is available from the preprint server.

